# Modelling future trajectories of obesity and body mass index in England

**DOI:** 10.1371/journal.pone.0252072

**Published:** 2021-06-02

**Authors:** Linda J. Cobiac, Peter Scarborough

**Affiliations:** Nuffield Department of Population Health, University of Oxford, Oxford, United Kingdom; Medical University of Vienna, AUSTRIA

## Abstract

**Background:**

Obesity is a leading risk for poor health outcomes in England. We examined best- and worst-case scenarios for the future trajectory of the obesity epidemic.

**Methods:**

Taking the last 27 years of Health Survey for England data, we determined both position and shape of the adult body mass index (BMI) distribution and projected these parameters 20 years forward in time. For the best-case scenario, we fitted linear models, allowing for a quadratic relationship between the outcome variable and time, to reflect a potential reversal in upwards trends. For the worst-case scenario, we fitted non-linear models that applied an exponential function to reflect a potential flattening of trends over time. Best-fitting models were identified using Monte Carlo cross-validation on 1991–2014 data, and predictions of population prevalence across five BMI categories were then validated using 2015–17 data.

**Results:**

Both linear and non-linear models showed a close fit to observed data (mean absolute error <2%). In the best-case scenario, the proportion of the population at increased risk (BMI≥25kg/m^2^) is predicted to fall from 66% in 2017 to 53% (95% confidence interval: 41% to 64%) in 2035. In the worst-case scenario, this proportion is likely to remain relatively stable overall– 64% (37% to 90%) in 2035 –but with an increasing proportion of the population at highest risk (BMI≥35kg/m^2^).

**Conclusions:**

While obesity prediction depends on chosen modelling methods, even under optimistic assumptions it is likely that the majority of the English population will still be at increased risk of disease due to their weight until at least 2035, without greater allocation of resources to effective interventions.

## Introduction

The last 30 to 40 years has seen a rapid rise in the prevalence of obesity worldwide. Initially a problem seen only in high income countries, obesity is now a leading risk factor for disease in all regions of the world [1]. Yet recent global modelling suggests that the rapid rises in obesity may be slowing in high-income, English-speaking countries, where epidemics first emerged [2].

Observation of obesity measures in the annual Health Survey for England suggest that this slowdown may be occurring in England [3]. Previous Foresight projection modelling of obesity, based on data collected through the 1990s and into the early 2000s, predicted large and sustained increases in obesity out to at least 2025 [4, 5], but more recent re-estimation of the models with post-2000s data suggests a flatter future trajectory in obesity levels [6].

In this study, we examine all available Health Survey for England data, from 1991 to 2017, fitting and validating models to simulate the changing distribution of body mass index (BMI) in the English population over time. This research aims to advance on the previous studies in three key ways.

First, we produce two sets of models reflecting two different interpretations of how current trends will propagate. All models that predict the future involve assumptions of how current trends will continue beyond the data collection period, but the impact of these assumptions on projections are rarely quantitatively tested. In the first set of models (‘linear’), we use polynomial regression equations to fit the slow-down in the increase of BMI that is observed in the data–when extrapolated these models can potentially result in a peak of BMI followed by accelerating decreases. In the second set of models (‘non-linear’) we apply an exponential function to constrain the increases in BMI over time–when extrapolated these models potentially result in ever slowing increases in BMI towards an asymptote. These two modelling approaches allow us to compare best-fitting ‘best-case’ and ‘worst-case’ future scenarios for BMI distributions in England.

Second, we take a two-step approach to regression: (1) modelling the *position* of the BMI distribution; and (2) modelling the *shape* of the BMI distribution, including the *position* as a covariate. While numerous models have been developed to examine future trends in mean BMI [7, 8], obesity prevalence [9, 10], or BMI categories [4, 5], these types of models cannot tell us about both the changing position and shape of the whole BMI distribution in the population. With our two-step approach, we aim to capture these multiple dimensions of change.

Third, rather than using all available data to fit each model, we take a Monte Carlo cross-validation approach to identify models that best fit the data, and additionally set data aside to validate predictions. By randomly splitting the data into separate groups for model fitting and model testing, and repeating this process many times, the Monte Carlo cross-validation approach reduces the probability of over-fitting models, in contrast to more traditional statistical approaches to model selection, such as a step-wise process comparing successive models using a likelihood ratio test.

## Methods

### BMI data

The Health Survey for England is an annual survey, designed to be representative of the English population. Data are available for download from the UK Data Service (beta.ukdataservice.ac.uk/datacatalogue/series/series?id=2000021). Surveys are currently available by single year of age from 1991 to 2014 and by five-year age group for 2015 to 2017. BMI was derived in all surveys from measures of height and weight recorded during a nurse visit. S1 Table in S1 File gives a summary of the survey data available for the study.

In this study of adult obesity, we focused on data for those aged 18 years or older. Given unusually low values of BMI reported in some years (e.g. 1998 and 1999) we restricted our modelling dataset to records with a BMI between 10 kg/m^2^ and 65 kg/m^2^. In effect, this removed 0.007% of records in the dataset.

For each year of survey data, we defined ten-year birth cohorts and determined the mean age and BMI within each cohort. Examination of the cohort data in Cullen and Frey graphs [11] suggested the data follow a lognormal, gamma or Weibull distribution (S1 and S2 Figs in S1 File). Comparison of these fitted distributions showed the lognormal distribution to be the best fit, based on the lowest Kolmogorov-Smirnov statistic (S2 Table in S1 File).

### Modelling approach

To model the position (*μ*) and shape (*σ*) of the BMI distribution for each of the two scenarios, we first defined a set of potential modelling equations including sex, age, year, and optional combinations of quadratic and interactions terms. There is good reason to expect that BMI will initially increase, but later decrease with increasing age [3], and that BMI may vary non-linearly through time, hence it was important to include the option of quadratic terms for these predictors. The key difference between the best- and worst-case scenarios was in the form of the relationship between the year and the outcome variable (*μ* or *σ*).

$$Linear:\;Y=x_{1}+x_{2}\;year+x_{3}\;{year}^{2}+x_{4}\;sex+x_{5}\;age+\cdots$$

$$Non‐Linear:\;Y=x_{1}-x_{2}\;{exp}^{-x_{3}\times year}+x_{4}\;sex+x_{5}\;age+\cdots$$

### Model fitting

The full range of potential models were trained and tested using data collected between 1991 and 2014, in a Monte Carlo cross-validation process. For each model scenario, we fitted the full range of potential model equations to a randomly-select 80% of the data (training set), and the best-fitting model was selected by comparing predictions from the fitted models with observations in the remaining 20% of the data (test set). Selection of the best-fitting model in the test data was based on the minimum root mean square error, which reflected the magnitude of deviation between predicted and observed values. This process was repeated 1000 times, and the optimal model was identified as the model with the highest probability of being the best-fitting model over the 1000 runs.

All analyses were performed in *R* (Version 3.5.1, www.r-project.org) using the *modelr* package (Version 0.1.2) for modelling.

### Model validation

We reserved survey data from years 2015 to 2017 for model validation. To validate the models, we first used the optimal *μ* and *σ* models from the cross-validation to predict the distribution of BMI across five broad categories: *Underweight* (<18.5 kg/m^2^), *Healthy weight* (18.5–24.99 kg/m^2^), *Overweight* (25–29.99 kg/m^2^), *Obese* (30–34.99 kg/m^2^) and *Very obese* (≥35 kg/m^2^). We then compared prevalence predictions, by sex and age group (18–24, 25–34, 35–44, 45–54, 55–64, 65–74, 75–84, 85+ years), with prevalence derived from observed data in the validation years, and estimated both the mean absolute error and root mean squared error (RMSE) for the linear and non-linear model sets.

### Prediction of future obesity

We used the validated models to predict the future prevalence of obesity in England, out to the year 2035. Numbers of obese individuals in the population were estimated using age- and sex-specific population projections (principal projection for England) from the Office for National Statistics [12].

## Results

Table 1 shows the optimal models of BMI *μ* and *σ*, and S3 Table in S1 File illustrates the underlying ranking process. Given a higher number of potential combinations of quadratic and interaction year terms to include in the equation set for the linear models, the cross-validation included a larger set of linear equations than non-linear equations. This meant that a wider selection of linear models were found to fit over the 1000 runs, and hence the overall frequency of selection of the highest ranked models was lower with the linear models than with the non-linear models. For example, the optimal linear model of *μ* was the best fit in 12% of runs while the optimal non-linear model of *μ* was the best fit in 51% of runs. However, testing with up to 2000 iterations did not alter the optimal model selection. The goodness of fit for the optimal linear and non-linear models was very similar (Table 2).

**Table 1:** Optimal linear and non-linear models.

	Term^a^	Estimate	Standard Error	t value	p value
Linear models					
μ	Intercept	2.972979986	0.004964579	598.83834	<0.001
	sex	0.014412279	0.001611178	8.945178832	<0.001
	age	0.011426627	0.00019795	57.72493726	<0.001
	year	0.002521202	1.18E-04	21.30066345	<0.001
	age^2^	-9.53E-05	1.78E-06	-53.4655526	<0.001
	year^2^	-1.21E-04	1.90E-05	-6.356827229	<0.001
σ	Intercept	-3.806540736	2.310486902	-1.647505871	0.100
	sex	-0.023975995	0.008183982	-2.929624628	0.004
	age	-3.82E-04	5.33E-04	-0.715965993	0.474
	year	0.002788139	2.76E-04	10.09602242	<0.001
	age^2^	-2.76E-06	4.48E-06	-0.615259472	0.539
	year^2^	-4.12E-05	4.15E-05	-0.990986204	0.322
	sex*age	-9.37E-04	3.38E-04	-2.774517921	0.006
	sex*age^2^	1.00E-05	3.05E-06	3.288031548	0.001
	age*year	-3.50E-05	4.62E-06	-7.590770738	<0.001
	age*year^2^	1.42E-06	7.09E-07	1.997336649	0.046
	μ	2.452629849	1.428785876	1.716583212	0.087
	μ^2^	-0.373191887	0.221068263	-1.688129641	0.092
Non-linear models^b^					
μ	a	3.005399647	0.008456307	355.4033207	<0.001
	b	0.024797114	0.005680661	4.365180892	<0.001
	year	0.090789991	0.015255284	5.951379925	<0.001
	sex	-0.002427327	0.009665538	-0.251132164	0.802
	age	0.011050057	2.75E-04	40.15140775	<0.001
	age^2^	-9.18E-05	2.46E-06	-37.2521751	<0.001
	sex*age	7.87E-04	3.95E-04	1.991540881	0.047
	sex*age^2^	-7.45E-06	3.56E-06	-2.091169767	0.037
σ	a	0.176626723	0.130804435	1.350311426	0.178
	b	-0.018412549	0.019345569	-0.951770854	0.342
	year	-0.048293744	0.044582701	-1.083239527	0.279
	sex	-0.023323632	0.009007944	-2.589229252	0.010
	age	5.21E-05	5.78E-04	0.090165218	0.928
	age^2^	-5.67E-06	4.88E-06	-1.162476146	0.246
	sex*age	-9.73E-04	3.70E-04	-2.62869905	0.009
	sex*age^2^	1.04E-05	3.34E-06	3.121223169	0.002
	μ	0.004283798	0.046904444	0.09133033	0.927

^a^ sex = 1 (male) or 0 (female); age in years; year = 0 in 2003.

^b^ Of the form Y ~ a—b * exp(-c * year) + ….

**Table 2:** Goodness of fit measures for the optimal models.

	Linear models		Non-linear models	
Measure	μ	σ	μ	σ
R-squared	0.916255	0.772483	–	–
Adjusted R-squared	0.915189	0.766017	–	–
Residual standard error	0.016086	0.013567	0.016043	0.01495
F-statistic	859.96	119.4522	–	–
p-value	<0.001	<0.001	–	–
Degrees of freedom	6	12	–	–
Log-likelihood	1084.661	1155.689	1086.752	1115.403
Akaike Information Criteria	-2155.32	-2285.38	-2155.5	-2210.81
Bayesian Information Criteria	-2127.4	-2233.52	-2119.6	-2170.92
Deviance	0.101691	0.07123	0.100631	0.087169
Residual degrees of freedom	393	387	391	390

When the optimal *μ* and *σ* models were combined to predict prevalence of *Underweight*, *Healthy weight*, *Overweight*, *Obese* and *Very obese*, by age and sex, in 2015 to 2017 (S3–S8 Figs in S1 File), the mean absolute error between predictions and observed values was just 1.58% for the linear models (RMSE: 0.0239) and 1.56% for the non-linear models (RMSE: 0.0241).

**Fig 1:**
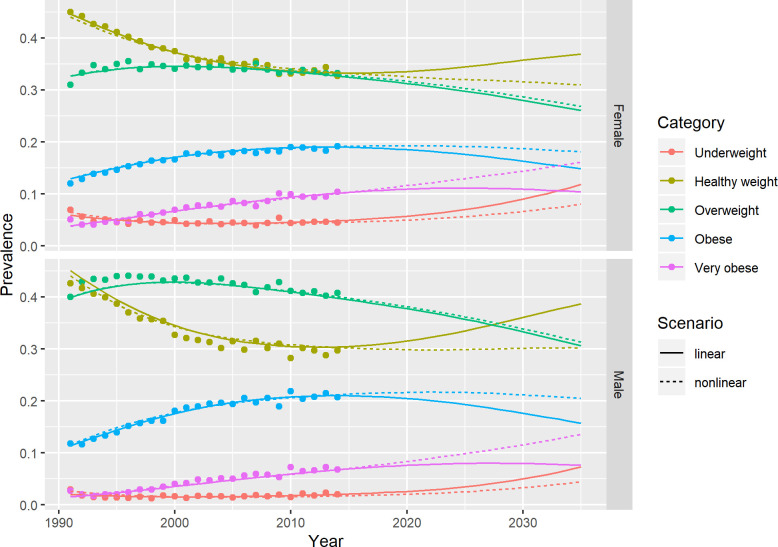
Comparison of linear and non-linear model predictions with data from the Health Survey for England.

Fig 1 shows the model predictions of prevalence for *Underweight*, *Healthy weight*, *Overweight*, *Obese* and *Very obese* out to the year 2035. The linear and non-linear models predictions are initially very similar, closely matching the observed data, but beyond 2017 they gradually diverge over time. In the best-case scenario, the linear models predict an accelerating shift in prevalence from the *Overweight*, *Obese* and *Very obese* end of the distribution back towards the *Healthy weight* and *Underweight* end of the BMI distribution. Whereas, in the worst-case scenario, the non-linear models predict more of a levelling-off in the *Healthy weight*, *Overweight*, and *Obese* categories, but increases in the *Underweight* and *Very obese* ends of the BMI distribution. The trends are similar by age and sex (S9 and S10 Figs in S1 File).

**Fig 2:**
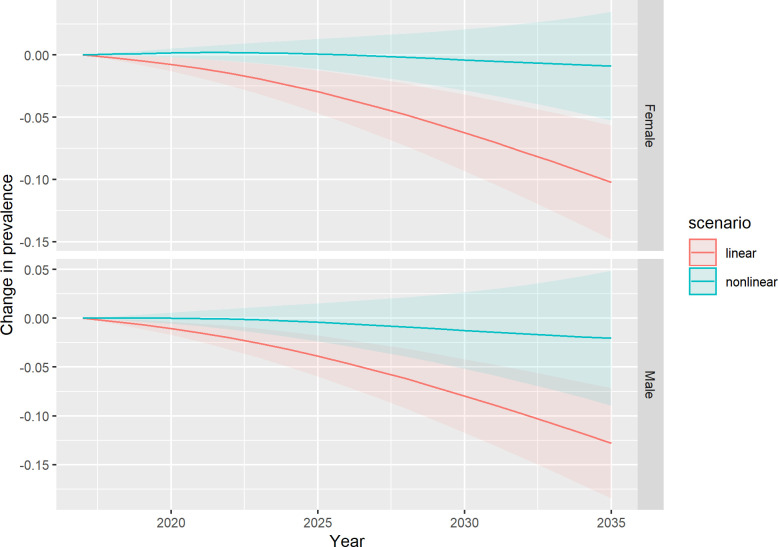
Predicted change in prevalence of the population at increased risk of disease (Overweight, Obese or Very obese).

**Fig 3:**
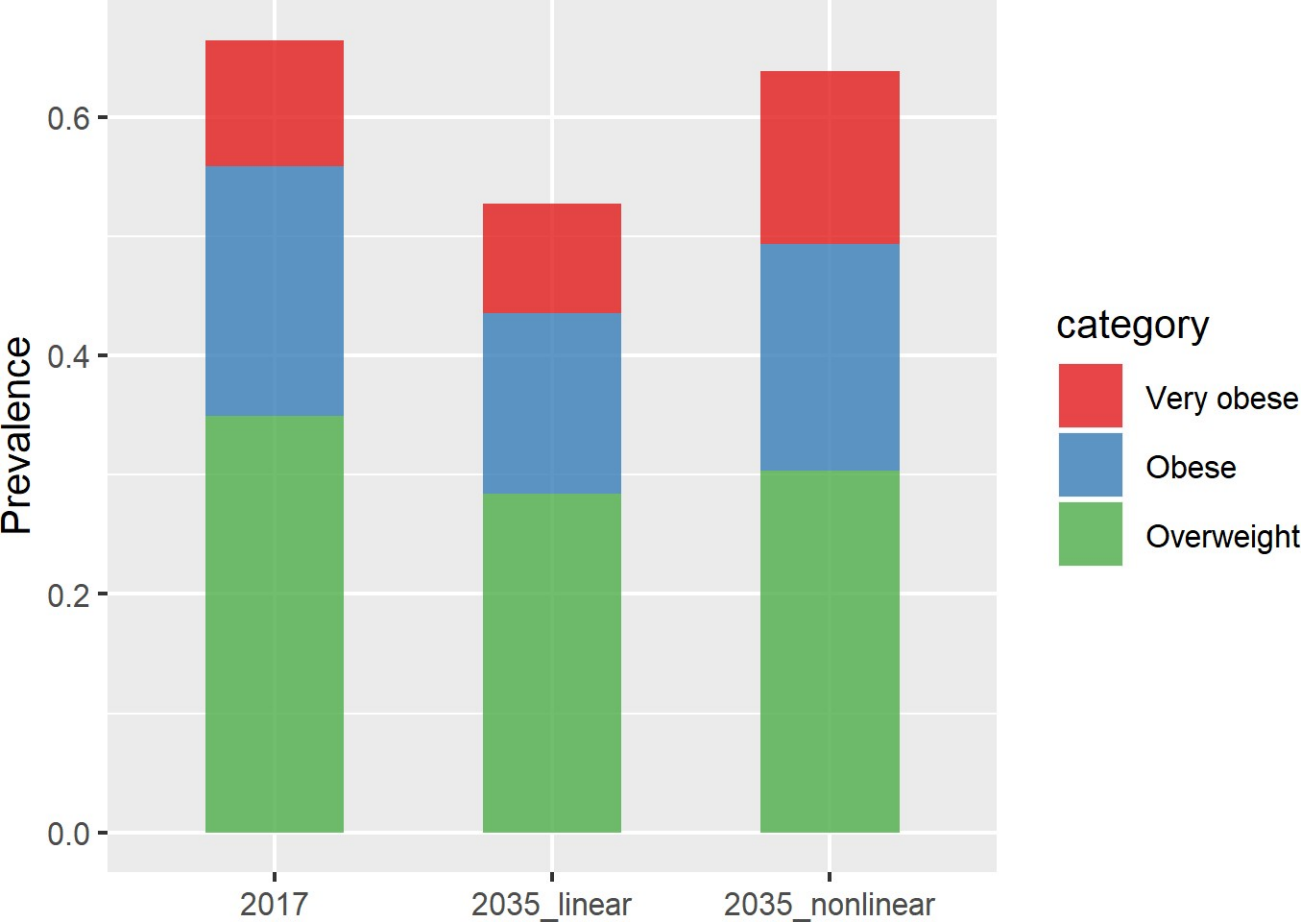
Total prevalence of the population at increased risk of disease (Overweight, Obese or Very obese) in 2017 and 2035.

Fig 2 shows the predicted change in the proportion of the population at increased risk of obesity-related disease (i.e. classified in our modelling as *Overweight*, *Obese* or *Very obese*). While the linear models predict a substantial decline in this proportion, the non-linear models predict no or little decrease on average. From an average value of 66% in 2017, our modelling predicts that by 2035 this proportion will at best reduce to 53% (41% to 64%) or at worst remain relatively stable at 64% (37% to 90%). In addition, in the worst-case scenario, the non-linear models predict that a larger proportion of those at risk are, on average, more likely to be at the *Very obese* end of the BMI spectrum, in comparison to predictions from the linear model (Fig 3 and Table 3).

**Table 3:** Predictions of obesity prevalence in 2035, with the linear and non-linear models.

		Linear models	Non-linear models
**Men**	Underweight	7.36% (2.96% to 11.8%)	4.87% (-1.45% to 11.2%)
	Healthy weight	38.5% (32.7% to 44.3%)	29.0% (25.1% to 32.9%)
	Overweight	30.7% (25.6% to 35.8%)	32.9% (17.3% to 48.4%)
	Obese	15.6% (12.6% to 18.6%)	20.1% (16.5% to 23.7%)
	Very obese	7.82% (3.27% to 12.4%)	13.2% (2.73% to 23.7%)
**Women**	Underweight	11.9% (7.04% to 16.7%)	8.09% (0.77% to 15.4%)
	Healthy weight	36.9% (32.3% to 41.6%)	30.4% (27.2% to 33.7%)
	Overweight	26.1% (22.3% to 29.8%)	27.8% (16.6% to 39.0%)
	Obese	14.7% (12.6% to 16.8%)	17.9% (14.8% to 21.1%)
	Very obese	10.4% (6.22% to 14.6%)	15.7% (6.53% to 24.9%)

NB. Values are mean and 95% confidence interval.

## Discussion

Our modelling of BMI in England illustrates how prediction of future obesity levels depends on the assumptions we make about how current trends will continue into the future beyond the data collection period. In the first set of models (linear), we used polynomial regression equations to reflect the slow-down in BMI increases observed in the Health Survey for England data, while in the second set of models (non-linear), we applied an exponential function to constrain the increases of BMI over time. Both sets of models achieved an excellent fit to the observed data. However, when extrapolated, the linear models predicted an accelerating decline in the proportion of the population at increased risk of disease (*Overweight*, *Obese* or *Very obese*), while the non-linear models predicted more of a stabilisation in this proportion overall, but with an accompanying shift in prevalence towards the higher-risk end of the BMI distribution. The two sets of models illustrated potential best- and worst-case scenarios for the future of obesity in England. Unfortunately, even in the best-case scenario, the modelling predicts that the majority of the English population will be at increased risk of disease due to excess body weight until at least 2035.

Our model predictions of obesity are generally lower than previous Foresight model predictions for England (S11 Fig in S1 File). Both modelling approaches are based on Health Survey for England data, but while we used all data collected between 1991 and 2014 (setting aside 2015–2017 for validation), the Foresight models have been estimated multiple times using different periods of data. Using data collected between 1993 and 2004, McPherson et al [4] predicted a prevalence of obesity (BMI ≥ 30kg/m^2^) of 47% for men and 36% for women by 2025, which is higher than either of our models estimate (best-case: 27% for men and 29% for women; worst-case: 32% for both men and women). However, more recent re-estimation of the same models using data collected between 2000 and 2013 [6], omitting earlier datasets when obesity levels were increasing most rapidly, predicted an obesity prevalence of just 34% for men and women combined. This prevalence is still higher than prediction with our best-case model (28%), but similar to prediction with our worst-case model (32%). Yet another estimation of the same models, using data collected between 2004 and 2014, predicted a rise in morbid obesity (BMI ≥ 40kg/m^2^) prevalence in England to 5% by 2025 and to 8% by 2035 [13]. This is higher than predictions with either of our models (best-case: 2.7% in 2025 and 3.0% in 2035; worst-case: 3.4% in 2025 and 5.6% in 2035).

Aside from the selection of different time-periods of data for fitting Foresight models, there are other differences in modelling approach. In the Foresight study [4], *Healthy*, *Overweight* and *Obese* categories of prevalence were modelled separately, with a post-hoc adjustment to ensure that modelled predictions of prevalence combined to 100% in any future year. Keaver et al [13] additionally modelled prevalence of *Morbid obesity* as a sub-set of the *Obese* category. We instead modelled the position of the BMI distribution, then modelled distribution shape, including position as a covariate.

There are a number of advantages in modelling multiple parameters of the BMI distribution. Our modelling of shape and position allowed prediction of future changes in the shape of the BMI distribution based in part on predicted changes in mean BMI. Taking a slightly different approach, Majer et al [14] fitted a Box-Cox power exponential distribution to model the location, scale, skewness and kurtosis of BMI. Later comparison of this four-parameter approach with a model of median BMI or logistic regression of obesity prevalence, showed better performance of the four-parameter model than the one-parameter models, particularly at the extreme tail of the BMI distribution [15]. In addition to overcoming the problem of prevalence predictions not adding up to 100% these methods of modelling multiple parameters of the BMI distribution allow for alternative cut-points or categorisations of obesity to be examined without a complete re-specification of models.

A key strength of this study is in the use of cross-validation to identify the optimal models. We examined models with a range of sex, age and year predictors, including quadratic and interaction terms. While we could have used a likelihood ratio test to examine significance in the addition of predictors, in a step-wise process, this would not necessarily have identified the model that best fits the data, nor would it have been possible to compare the relative fit of our linear and non-linear models. By randomly splitting the data into separate groups for model fitting and model testing, the cross-validation process reduces the probability of over-fitting. In this study, we chose to repeat the fitting and testing process 1000 times, then ranked the models in order of frequency of selection as the best fitting model, and selected the highest ranked model as the optimal fit. Alternative selection processes, such as estimating the standard error in the mean square error across the multiple runs, then choosing the simplest model that is within one standard error of the minimum cross-validation error [16], have also been proposed.

The results of the BMI modelling have important implications for population well-being and funding for treatment of obesity-related diseases in England. Even under our best-case scenario, the majority of the English population are still predicted to be living at an unhealthy weight by 2035. England’s former Chief Medical Officer has recognised obesity as a key driver of poor health, and has identified a range of interventions to address the problem [17]. A Soft Drinks Industry Levy, implemented in April 2018, received strong support from the UK population [18], and has already been associated with a 39 percentage point fall in the number of soft drinks with sugar levels high enough to be eligible for the levy [19]. Sugar-reduction targets for a range of foods, a 9pm watershed on television advertising of unhealthy foods, and programs to address physical activity in schools and local areas have also been proposed.

It is important to note that our models inherently assume the status quo with regard to influences on body mass in the population. Diversion from current trends in access to and affordability of healthy foods or opportunities for physical activity will shift the population away from the predicted pathways. When new Health Survey for England data are released, further research will be needed to examine the potential effects of the new and emerging interventions on the predicted trajectories of obesity.

Further research could also examine the influence of deprivation on obesity trends in England. A higher prevalence of obesity is associated with higher levels of social and economic deprivation [20]. Previous analyses of weight surveillance data from primary school-age children in England, suggested an increasing inequality in BMI between the most and least deprived areas [21]. While studies of obesity trends in adults in Health Survey for England data to 2004, did not find significant differentials when examined by social class [4, 22], further analysis including more recent datasets may be warranted.

## Conclusion

In this modelling study of BMI distributions, drawing on 27 years of Health Survey for England data, combined models of the position and shape of the BMI distribution accurately predicted prevalence across multiple levels or categories of obesity. Both linear polynomial regression and non-linear exponential models showed a good fit to observed data, but provided very different predictions of future BMI trajectories. We recommend that modellers consider using more than one technique in BMI projection modelling to reflect the range of potential best- and worst-case outcomes.

For England, predictions with fitted models suggest that even under the more optimistic modelling assumptions, the majority of the adult population are still likely to be at increased risk of disease due to their weight until at least 2035. Further intervention to address access to and availability of healthy foods and opportunities for physical activity, will be needed if England is to avert the consequent burden to population health and costs to the National Health Service for treating obesity-related disease.

## Supporting information

S1 File (PDF)
