# Supplementary material for: Modelling future trajectories of obesity and body mass index in England
Source: PLoS One. 2021 Jun 2;16(6):e0252072. doi: 10.1371/journal.pone.0252072 (PMC8172072; doi:10.1371/journal.pone.0252072)
Supplement: S1 File — (PDF) [file pone.0252072.s001.pdf]

S1 File

S1 Table. Summary of the BMI data in the Health Survey for England surveys.

Survey			Age[a]		BMI[b]				
Year	N	% female	Min	Max	N	Mean	SD	Min	Max
1991	3,242	54%	16	98	3,075	25.6	5.0	14.7	74.2
1992	4,416	49%	16	98	4,203	25.7	4.5	14.0	63.8
1993	17,687	52%	16	99	15,758	25.8	4.4	13.7	72.5
1994	15,809	55%	16	102	14,831	25.9	4.5	14.1	63.1
1995	19,788	53%	2	100	18,133	24.5	5.3	11.0	55.9
1996	20,328	53%	2	102	19,038	24.6	5.4	11.5	53.9
1997	15,546	53%	2	95	14,502	22.6	5.9	10.1	65.9
1998	19,654	54%	2	98	17,741	24.8	5.6	3.8	55.0
1999	9,640	53%	2	96	8,549	24.9	5.7	3.2	63.6
2000	12,413	58%	2	107	9,005	25.0	5.7	8.6	61.3
2001	19,640	55%	0	99	16,927	25.2	5.8	5.9	57.0
2002	18,398	53%	0	97	15,983	22.8	5.9	8.0	59.4
2003	18,553	54%	0	99	16,062	25.4	5.9	10.6	70.7
2004	8,354	55%	0	98	6,803	25.6	5.8	12.2	57.4
2005	13,297	54%	0	100	10,823	25.3	6.0	10.6	58.8
2006	21,399	53%	0	99	18,126	24.3	6.3	1.9	77.0
2007	14,386	52%	0	99	12,332	22.6	6.2	4.1	67.0
2008	22,619	54%	0	98	19,045	24.4	6.3	9.9	65.8
2009	8,602	27%	0	96	3,830	23.4	6.5	10.7	64.8
2010	14,112	53%	0	98	11,509	23.9	6.6	3.6	81.8
2011	10,617	54%	0	100	8,376	25.9	6.1	8.3	65.3
2012	10,333	54%	0	98	8,199	25.9	6.1	11.8	62.9
2013	10,980	54%	0	104	8,799	25.9	6.2	12.4	63.1
2014	10,080	54%	0	90	8,336	26.0	6.2	11.3	66.7
2015	13,748	53%	2-4	85+	11,260	23.8	6.7	10.0	69.1
2016	10,067	54%	2-4	85+	7,808	26.0	6.4	8.5	63.4
2017	9,982	55%	2-4	85+	7,803	26.3	6.6	11.3	61.7

[a] Age reported in age groups from 2015

[b] Summary statistics exclude records with BMI less than 1 kg/m^2

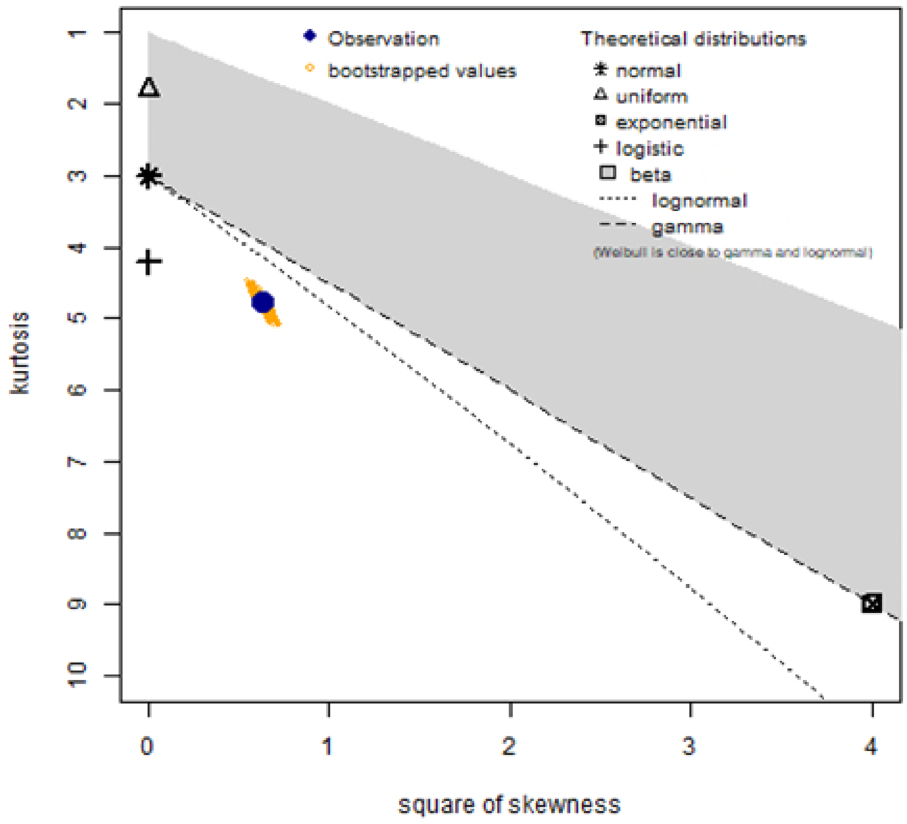

S1 Fig. Cullen and Frey graph for men

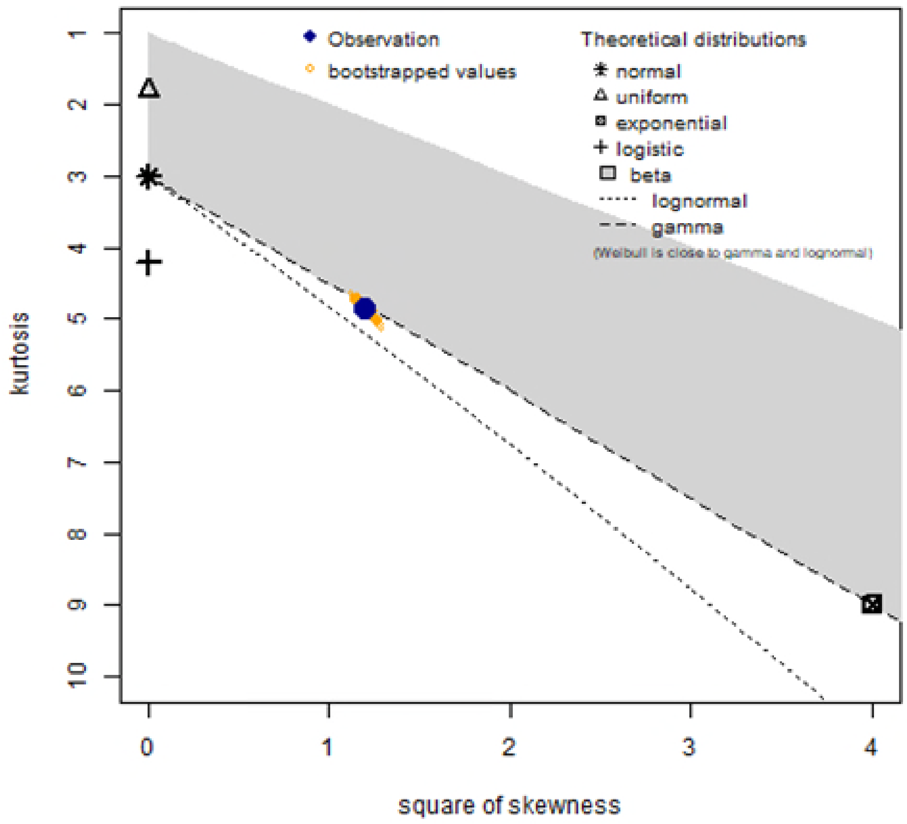

S2 Fig. Cullen and Frey graph for women

S1 Table. Kolmogorov-Smirnov test indicating best fit (lowest value) for the lognormal distribution in men and women

Distribution	Kolmogorov-Smirnov test	
	Men	Women
Normal	0.047598	0.077103
Lognormal	0.017776	0.038945
Gamma	0.026365	0.052031
Weibull	0.081307	0.103481

S2 Table. Ranking of models based on frequency of model selection in the 1000 runs (top five shown)

		Frequency	Rank
Linear models			
μ	μ ~ sex + age + year + age^2 + year^2	123	1
	μ ~ sex + age + year + age^2 + year^2 + sex*age + sex*age^2	118	2
	μ ~ sex + age + year + age^2 + year^2 + sex*age + sex*age^2 + age*year	88	3
	μ ~ sex + age + year + age^2 + year^2 + age*year + age*year^2 + age^2_year + age^2_year^2	80	4
	μ ~ sex + age + year + age^2 + year^2 + sex*age + sex*age^2 + age*year + age^2_year	77	5
σ	σ ~ sex + age + year + age^2 + year^2 + sex*age + sex*age^2 + age*year + age*year^2 + μ + μ^2	99	1
	σ ~ sex + age + year + age^2 + year^2 + sex*age + sex*age^2 + age*year + μ + μ^2	88	2
	σ ~ sex + age + year + age^2 + year^2 + sex*age + sex*age^2 + age*year + age*year^2 + μ	84	3
	σ ~ sex + age + year + sex*age + age*year + μ + μ^2	70	4
	σ ~ sex + age + year + age^2 + year^2 + sex*age + sex*age^2 + age*year + age*year^2 + age^2_year + age^2_year^2 + μ + μ^2	45	5
Non-linear models			
μ	μ ~ a - b exp(-c year) + d sex + e age + f age^2 + g sex*age + h sex*age^2	514	1
	μ ~ a - b exp(-c year) + d sex + e age + f age^2	401	2
	μ ~ a - b exp(-c year) + d sex + e age + f age^2 + g sex*age	85	3
	μ ~ a - b exp(-c year) + d sex + e age	0	–
	μ ~ a - b exp(-c year) + d sex + e age + g sex*age	0	–
σ	σ ~ a - b exp(-c year) + d sex + e age + f age^2 + g sex*age + h sex*age^2 + i μ	457	1
	σ ~ a - b exp(-c year) + d sex + e age + g sex*age + i μ	250	2
	σ ~ a - b exp(-c year) + d sex + e age + i μ	156	3
	σ ~ a - b exp(-c year) + d sex + e age + f age^2 + i μ	90	4
	σ ~ a - b exp(-c year) + d sex + e age + f age^2 + g sex*age + i μ	47	5

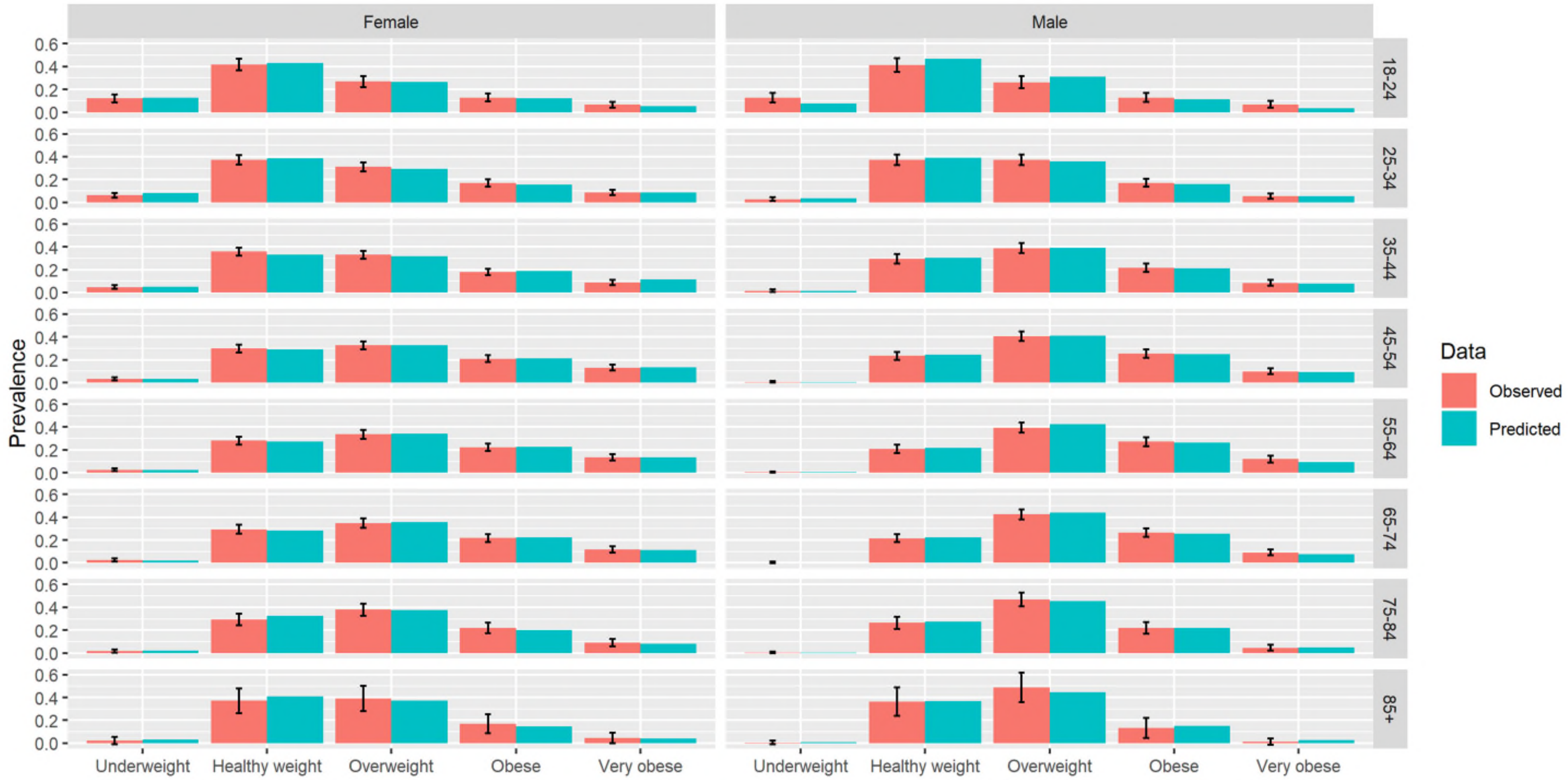

S3 Fig. Comparison of best-case linear model predictions with observations from the Health Survey for England in 2015

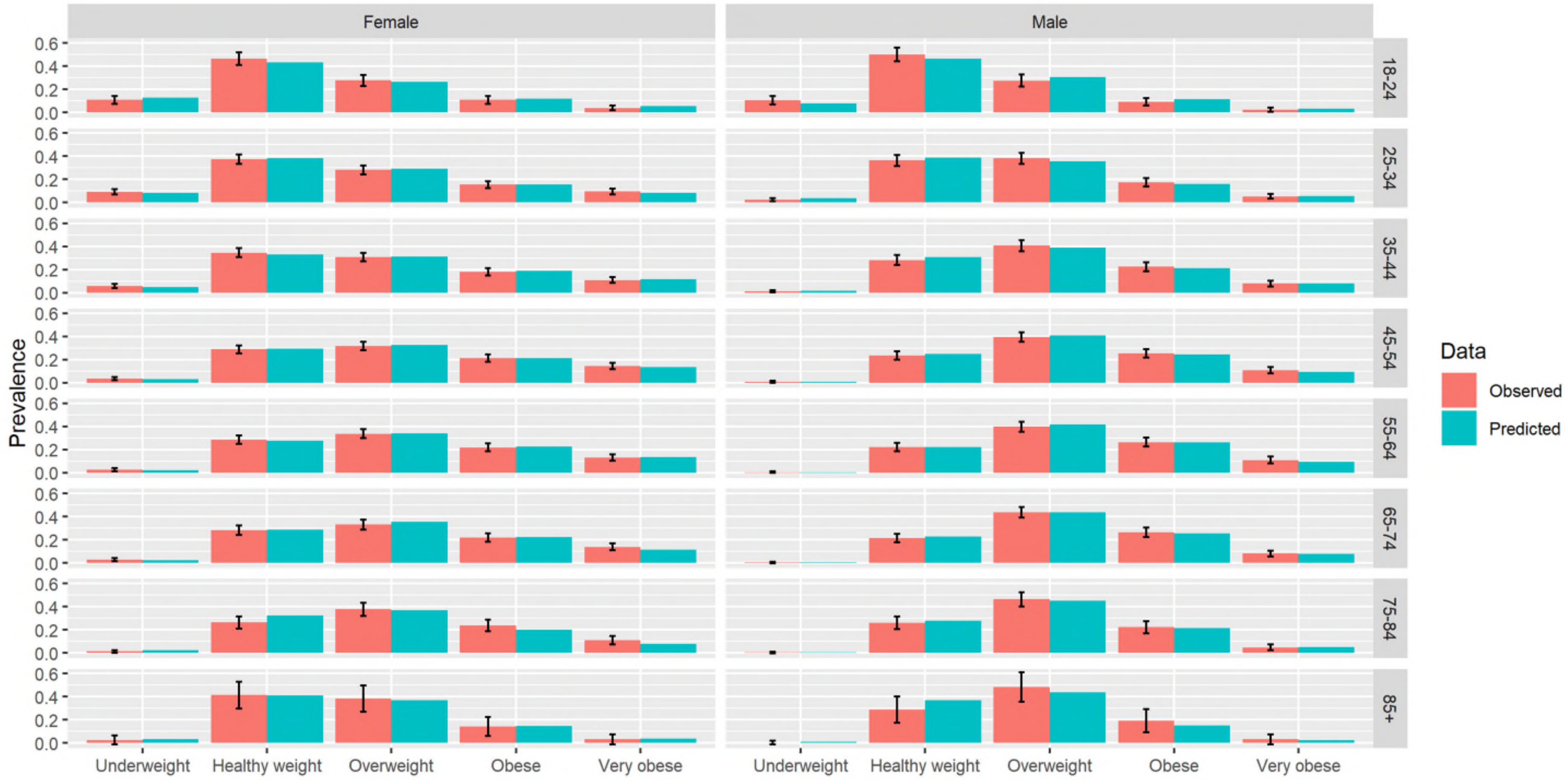

S4 Fig. Comparison of best-case linear model predictions with observations from the Health Survey for England in 2016

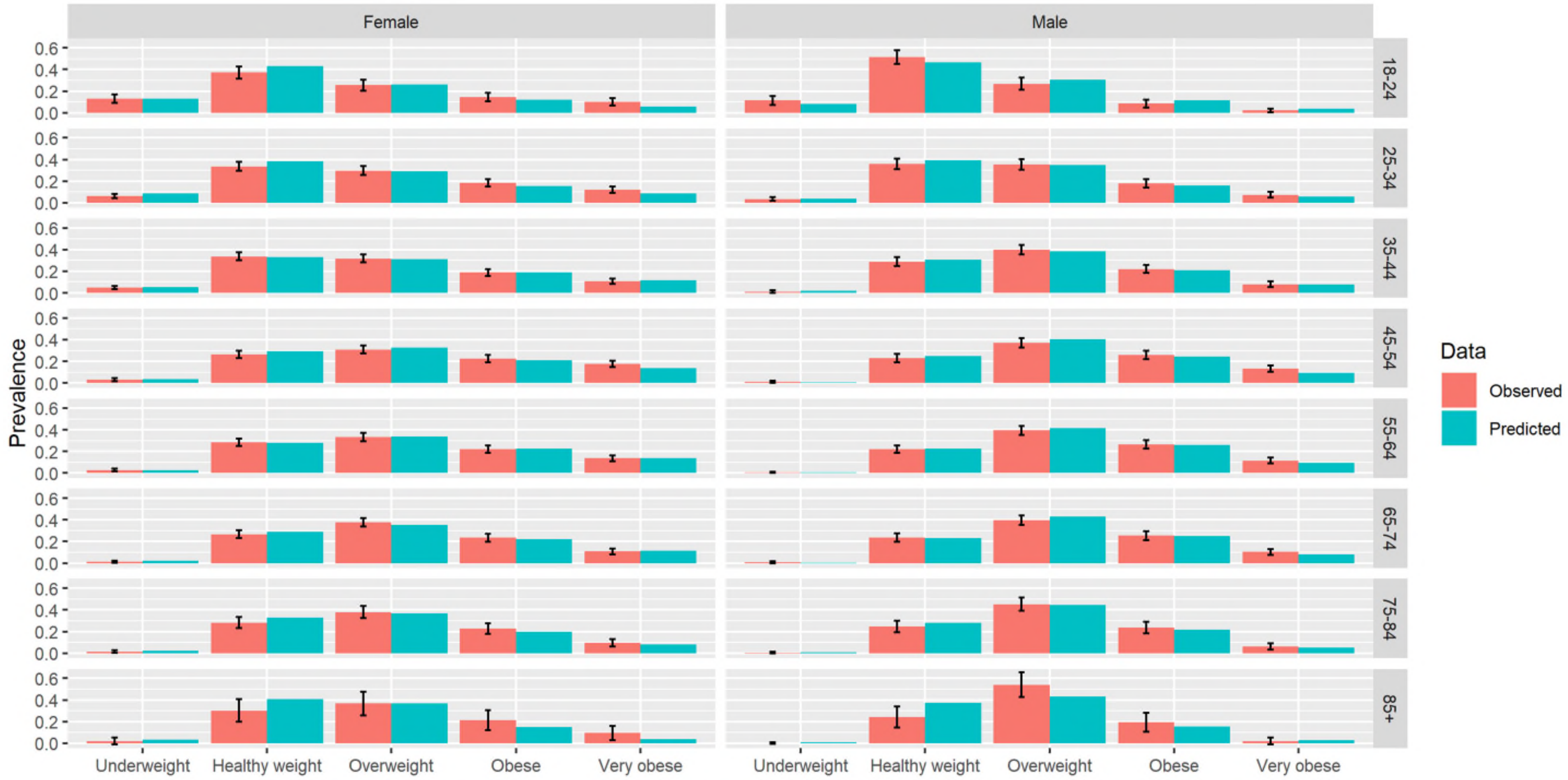

S5 Fig. Comparison of worst-case non-linear model predictions with observations from the Health Survey for England in 2017

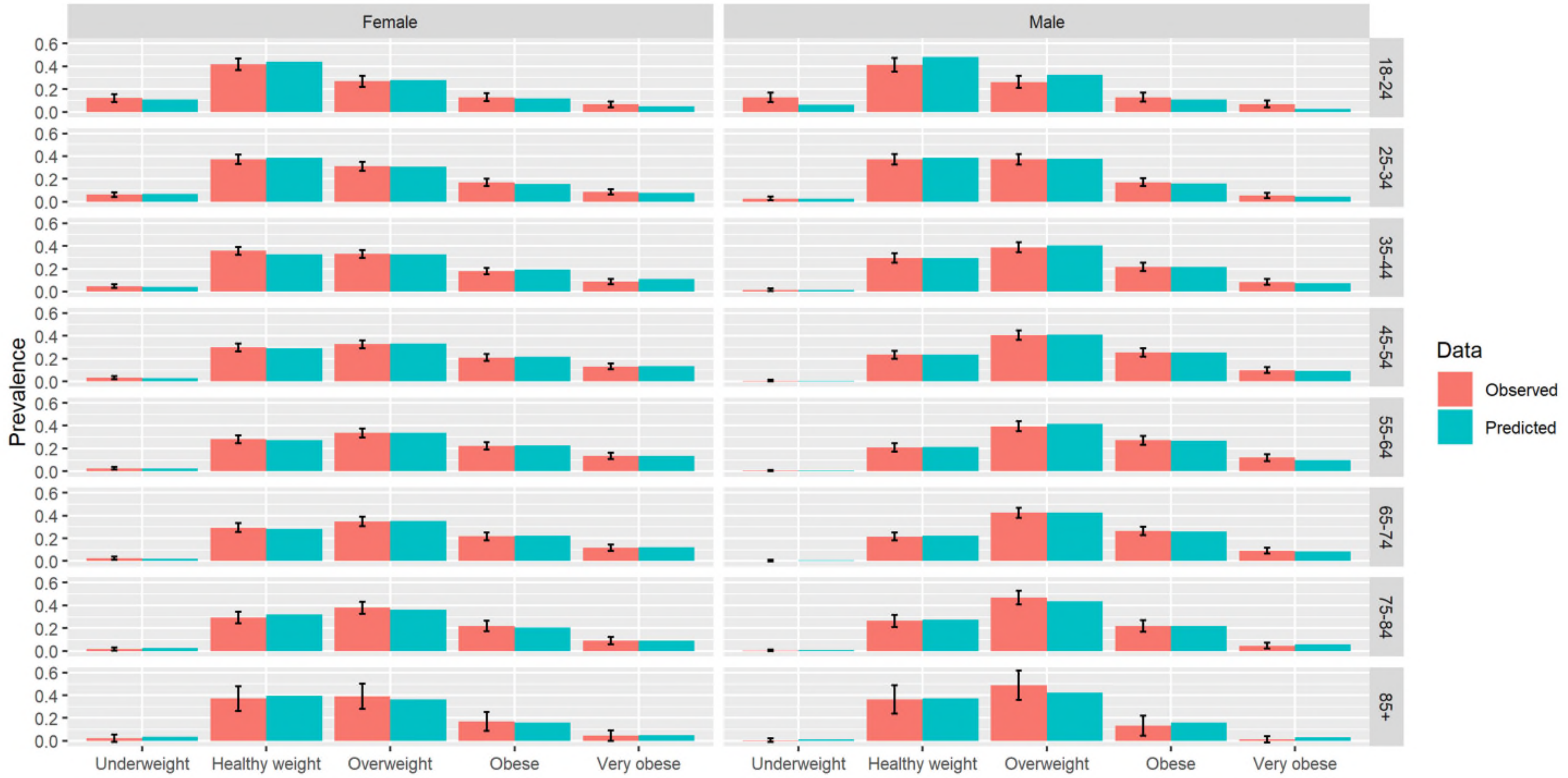

S6 Fig. Comparison of worst-case non-linear model predictions with observations from the Health Survey for England in 2015

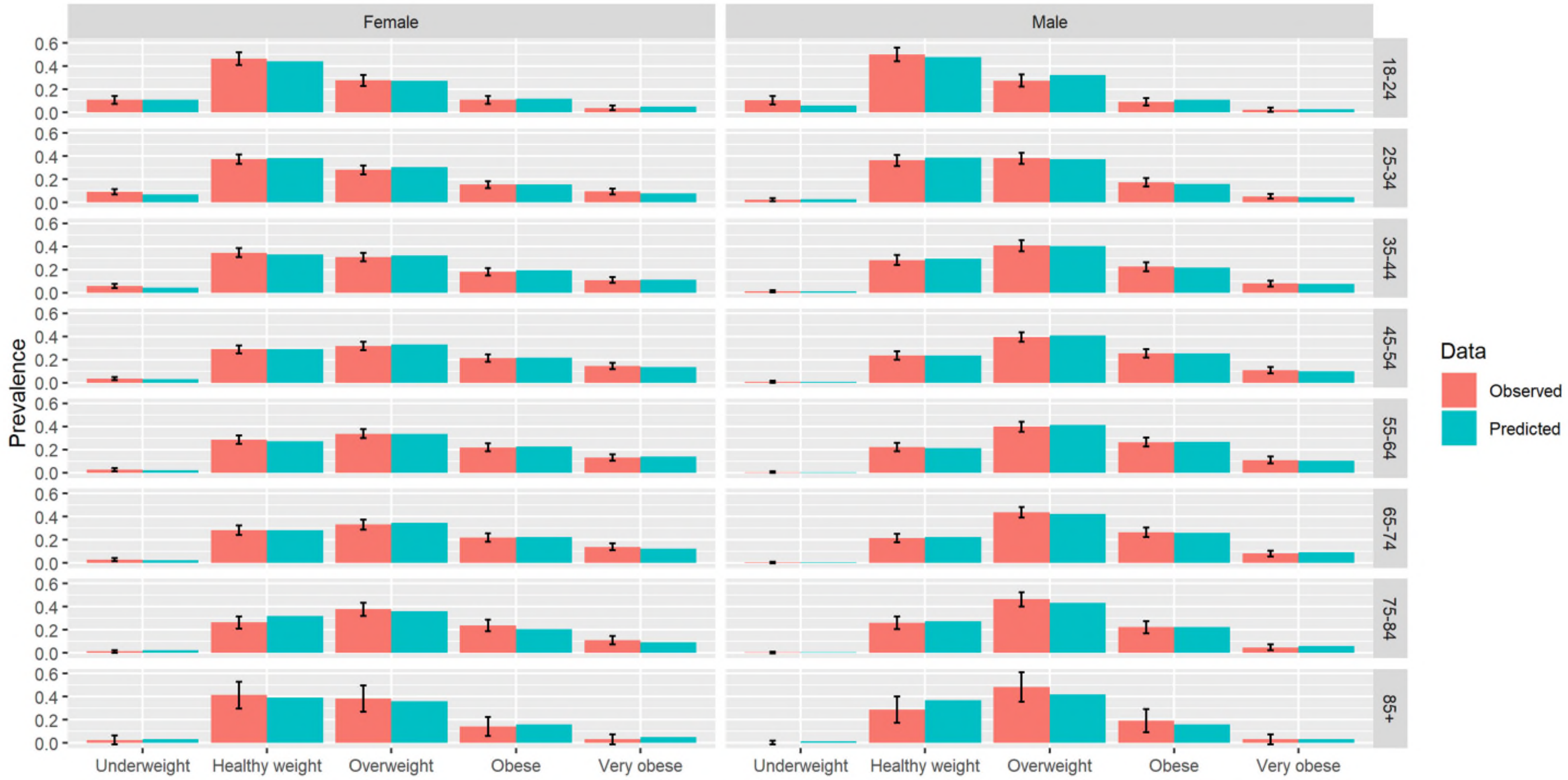

S7 Fig. Comparison of worst-case non-linear model predictions with observations from the Health Survey for England in 2016

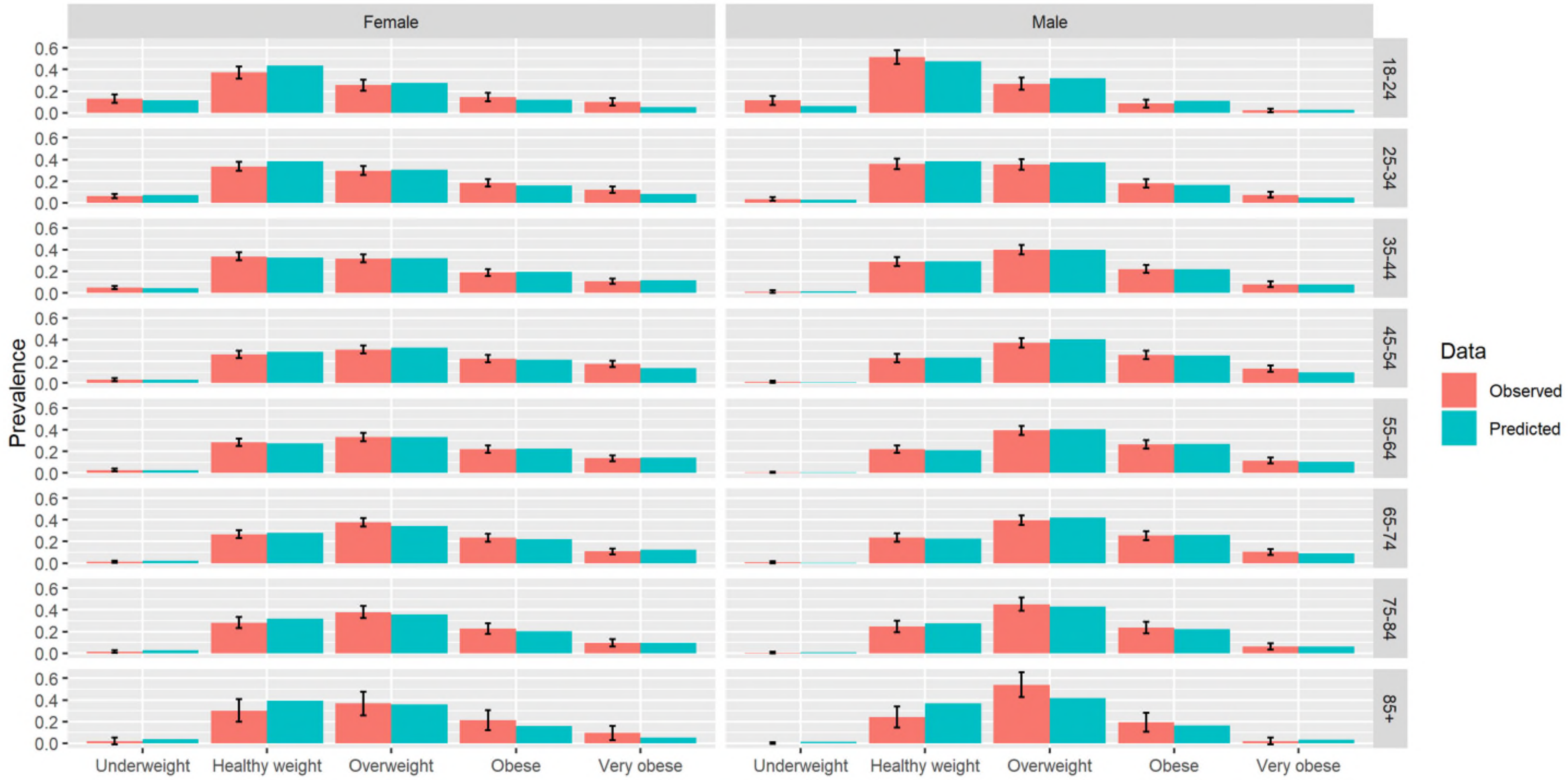

S8 Fig. Comparison of worst-case non-linear model predictions with observations from the Health Survey for England in 2017

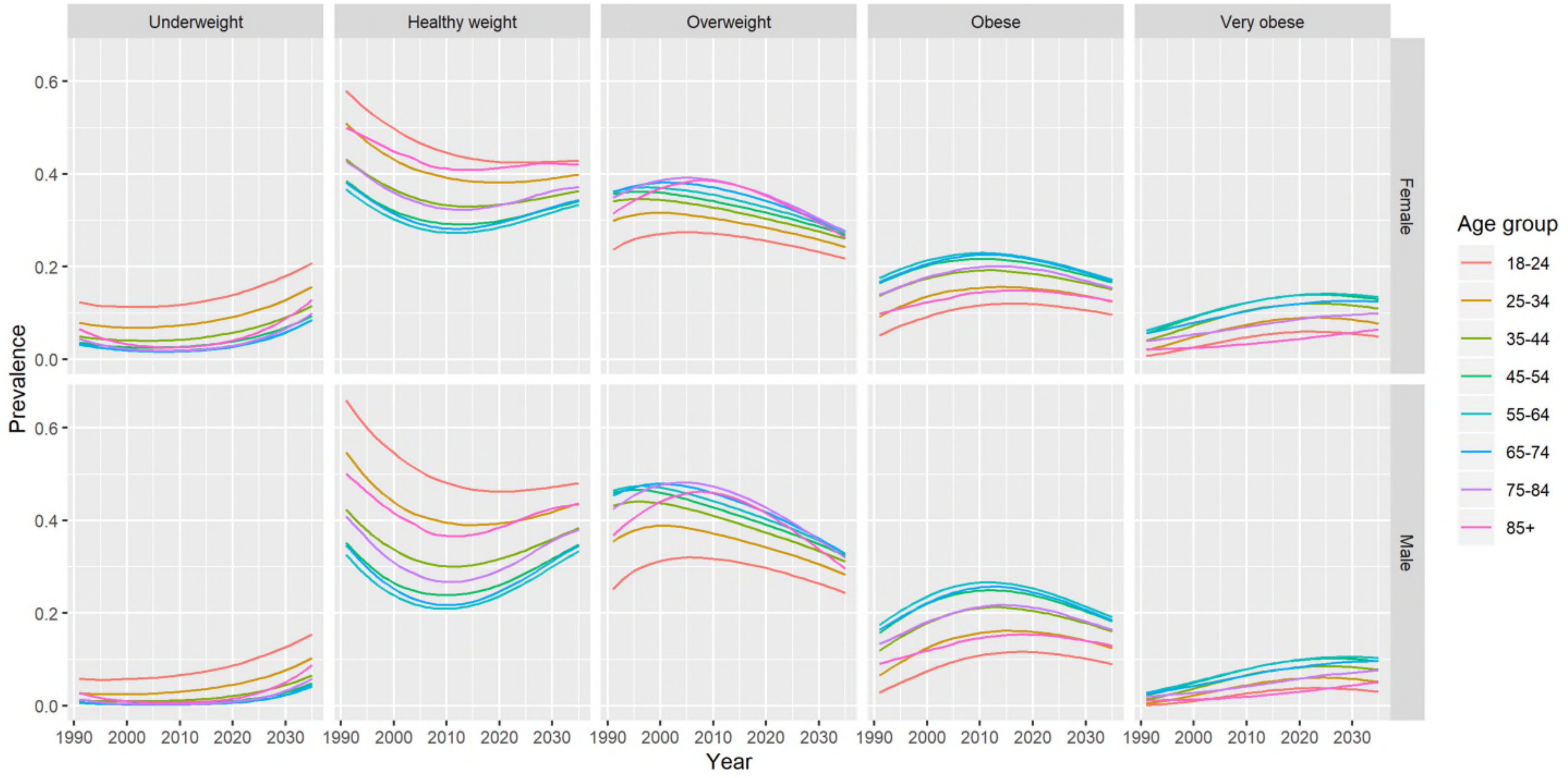

S9 Fig. Predicted trend in prevalence, by age and sex, with the linear models.

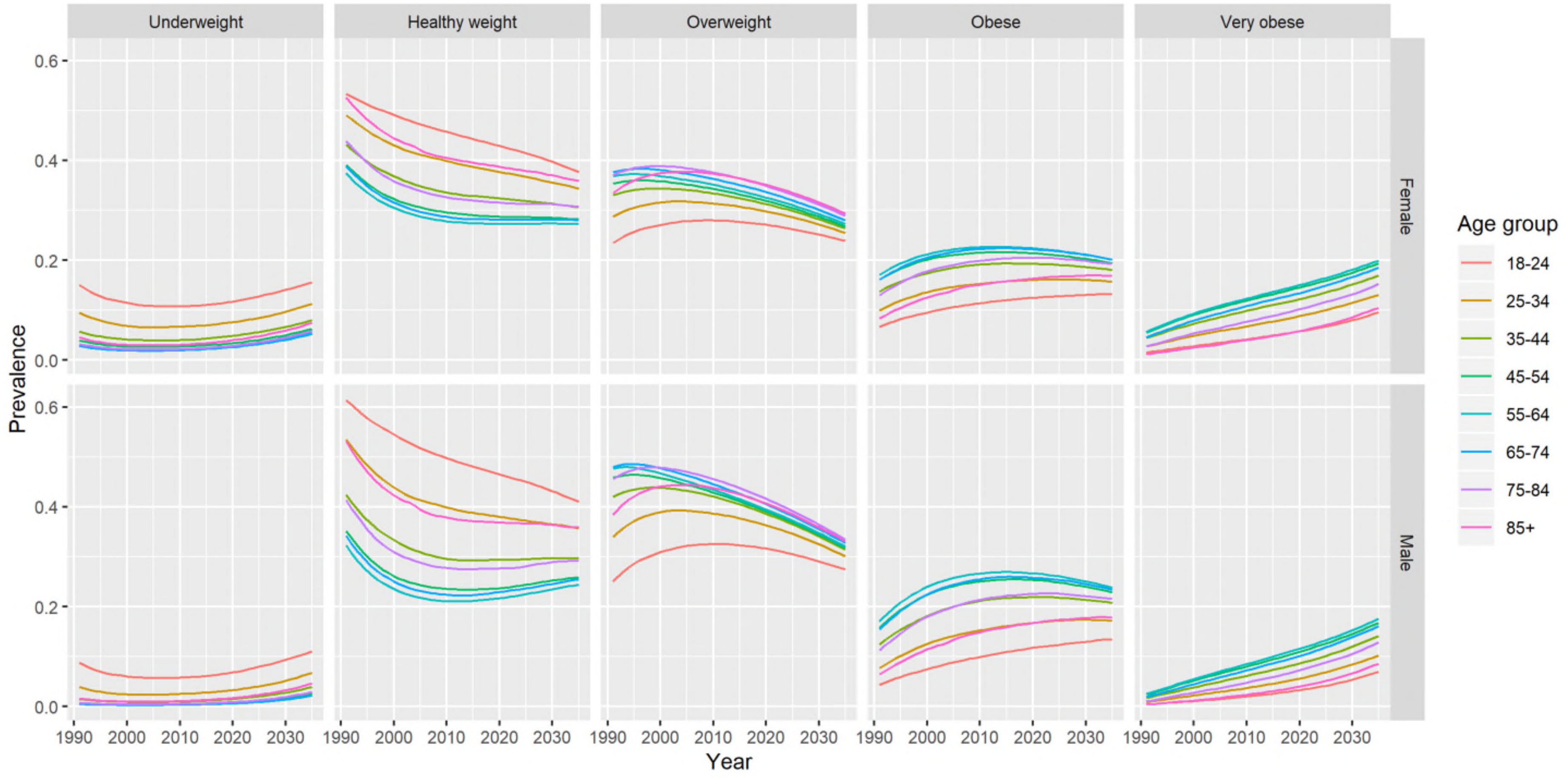

S10 Fig. Predicted trend in prevalence, by age and sex, with the non-linear models.

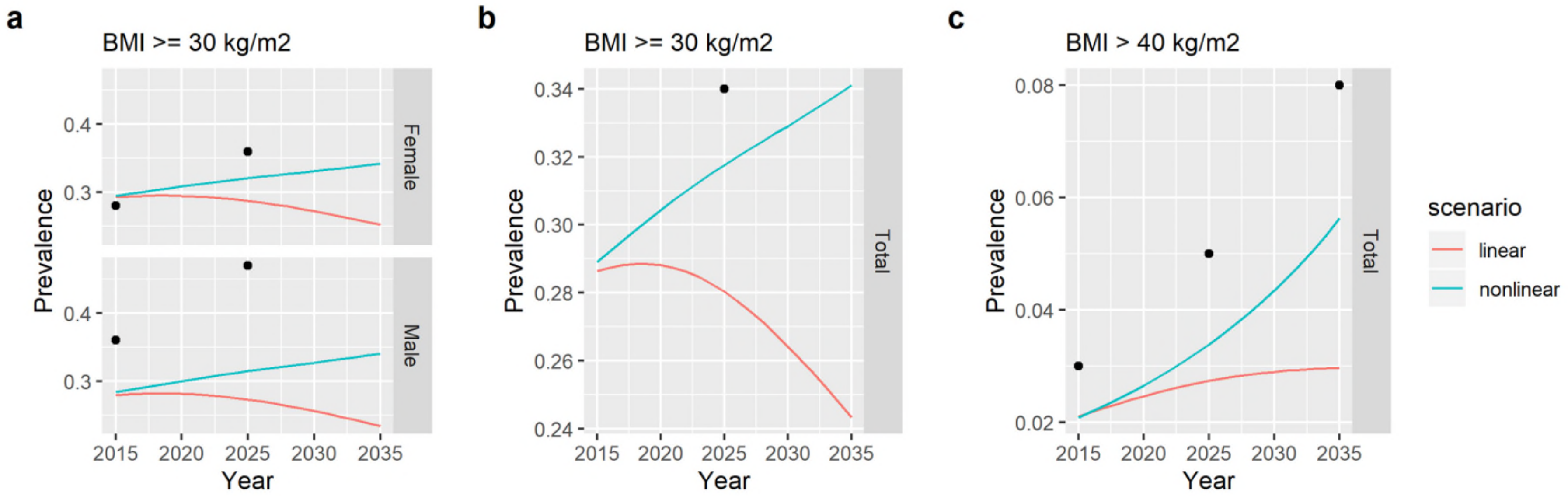

S10 Fig. Comparison of linear and non-linear model predictions with previous Foresight predictions of obesity (black points in the graphs represent point-estimates reported in: (a) McPherson et al [4]; (b) Pineda et al [6]; and (c) Keaver et al [13]).
